# EPINETLAB: A Software for Seizure-Onset Zone Identification From Intracranial EEG Signal in Epilepsy

**DOI:** 10.3389/fninf.2018.00045

**Published:** 2018-07-11

**Authors:** Lucia R. Quitadamo, Elaine Foley, Roberto Mai, Luca de Palma, Nicola Specchio, Stefano Seri

**Affiliations:** ^1^School of Life and Health Sciences, Aston Brain Centre, Aston University, Birmingham, United Kingdom; ^2^Claudio Munari Epilepsy Surgery Center, Niguarda Hospital, Milan, Italy; ^3^Pediatric Neurology Unit, Department of Neuroscience and Neurorehabilitation, Bambino Gesù Children’s Hospital, Rome, Italy; ^4^Department of Clinical Neurophysiology, The Birmingham Women’s and Children’s Hospital NHS Foundation Trust, Birmingham, United Kingdom

**Keywords:** EEGLAB, epilepsy, high-frequency oscillations, seizure-onset zone, iEEG, stereo-EEG

## Abstract

The pre-operative workup of patients with drug-resistant epilepsy requires in some candidates the identification from intracranial EEG (iEEG) of the seizure-onset zone (SOZ), defined as the area responsible of the generation of the seizure and therefore candidate for resection. High-frequency oscillations (HFOs) contained in the iEEG signal have been proposed as biomarker of the SOZ. Their visual identification is a very onerous process and an automated detection tool could be an extremely valuable aid for clinicians, reducing operator-dependent bias, and computational time. In this manuscript, we present the EPINETLAB software, developed as a collection of routines integrated in the EEGLAB framework that aim to provide clinicians with a structured analysis pipeline for HFOs detection and SOZ identification. The tool implements an analysis strategy developed by our group and underwent a preliminary clinical validation that identifies the HFOs area by extracting the statistical properties of HFOs signal and that provides useful information for a topographic characterization of the relationship between clinically defined SOZ and HFO area. Additional functionalities such as inspection of spectral properties of ictal iEEG data and import and analysis of source-space magnetoencephalographic (MEG) data were also included. EPINETLAB was developed with user-friendliness in mind to support clinicians in the identification and quantitative assessment of HFOs in iEEG and source space MEG data and aid the evaluation of the SOZ for pre-surgical assessment.

## Introduction

Every year 2.4 million people are diagnosed with epilepsy; it has been estimated that 25% of them respond inadequately to pharmacological treatment and could therefore be potential candidates to resective surgery (Banerjee et al., 2009; Kwan et al., 2011). An accurate delineation of the epileptogenic zone (EZ), i.e., the area of cortex that is necessary and sufficient for initiating seizures and whose removal is necessary for complete abolition of seizures, is fundamental for a positive surgical outcome. This process relies on the convergence of clinical information with the results of a wide range of investigative tools and techniques (Rosenow and Lüders, 2001; Engel et al., 2013). In the last decade, high-frequency oscillations (HFOs) in the intracranial EEG (iEEG) have gained increasing interest as potential biomarkers of epileptogenesis, having shown close spatial relationship with the seizure-onset zone (SOZ) in patients with focal epilepsy (Jacobs et al., 2008; Worrell and Gotman, 2011; Zijlmans et al., 2012). SOZ is the area of the cortex from which seizures originate and is currently used as a surrogate of the EZ in the clinical practice.

The visual detection of HFOs in multichannel long-term iEEG is a challenging task even for an expert operator and this has so far somewhat limited a more widespread use in clinical practice. This limitation has driven recent interest in developing detection algorithms (Blanco et al., 2010; Dümpelmann et al., 2012; Burnos et al., 2014; Fedele et al., 2016; Gliske et al., 2016) and implementing these in time-efficient analysis tools that require minimal human supervision (Navarrete et al., 2016).

In this manuscript, we present EPINETLAB, a multi-graphic user interface (GUI) set of Matlab functions developed in the context of the **EPI**leptic **NET**works project (EPINET^1^), a EU-funded initiative focused on the development of tools for the detection of HFOs in iEEG and source-space magnetoencephalographic (MEG) data (Foley et al., 2017; Quitadamo et al., 2017) and on their application to improve the delineation of the SOZ. The tool was developed as a plugin for EEGLAB (Delorme and Makeig, 2004), under the GNU Public License version 3.0 and can be found at the following link: https://github.com/quitadal/EPINETLAB. The choice of implementation as an EEGLAB extension was justified by the wide acceptance of this platform in the neurophysiology community as a tool for EEG and Evoked Potentials data analysis. EEGLAB main advantages include:

(1) The ability to import data from a wide range of file formats which can be easily extended to others not yet supported with purpose-developed Matlab code;
(2) a wide range of functions for pre-processing of brain signals such as artifact rejection, independent component analysis, signal averaging, and spectral analysis including time-frequency decomposition;
(3) the extensible and open-source nature of this platform, which is supported by a strong research team and is enriched by plugins developed in laboratories around the world.

EPINETLAB was designed to provide an easy-to-use tool to investigate the spatial and time-frequency properties of HFOs, to identify the iEEG channels with the highest HFO rate (which we will refer to as the “HFO area”) and to provide measures to support the evaluation of the spatial distribution of the HFO area with that of the SOZ identified in the presurgical workup. The toolbox is supported by detailed documentation of each step of the analysis pipeline; parameters for the analyses can be set in GUIs designed with user-friendliness in mind. Moreover, the platform allows analysis of multiple files in a single process and the implementation of a robust channel reduction methodology was designed to reduce computational load and subject-dependent errors. An addition that is not available in other tools released so far in the public domain (Navarrete et al., 2016) is the possibility to load, process, and analyze MEG data in signal and source-space, thus providing the possibility to evaluate the concordance between the source locations of HFOs recorded from pre-operative MEG studies and those identified in iEEG recordings. Each function in the tool underwent a rigorous beta-testing phase with neurophysiology clinical scientists (EEG Technologists) and clinical neurophysiologists, to simulate real-life operator-dependent situations and minimize unexpected software termination. In this manuscript, we present the main features of EPINETLAB as well as examples of HFO detection and SOZ identification from an iEEG clinical dataset.

## Materials and Methods

### Tool Validation

The initial validation was performed on the iEEG of 12 patients (6 female, mean age ± SD: 21.25 ± 11.34 years) who underwent presurgical evaluation either at the Niguarda Hospital (NIG), Milan, Italy or at the Birmingham Women’s and Children’s Hospital (BCH) in Birmingham, United Kingdom. Patients’ information is reported in **Table 1**.

**Table 1:** Patients’ information.

Patient	Pathology	Institution	Implantation type	Engel class
1	Gliosis	NIG	SEEG	Ia
2	Type IIa Focal cortical dysplasia	NIG	SEEG	Ia
3	Type IIb Focal cortical dysplasia	NIG	SEEG	II
4	Type IIa Focal cortical dysplasia	NIG	SEEG	Ia
5	Type IIa Focal cortical dysplasia	NIG	SEEG	Ia
6	Type IIb Focal cortical dysplasia	BCH	SEEG	Ia
7	Type IIa Focal cortical dysplasia	BCH	SEEG + GRID	Ia
8	Ganglioglioma Grade 1	BCH	GRID	Ia
9	Hippocampal Sclerosis	BCH	SEEG	Ia
10	Meningioangiomatosis	BCH	GRID	Ia
11	Type Ib Focal cortical dysplasia	BCH	GRID	Ia
12	Pilocytic Astrocytoma	BCH	GRID	Ia

*Engel’s classification: I, free from disabling seizures (Ia, completely seizure free since surgery); II, Rare disabling seizures; III, Worthwhile improvement; IV, No worthwhile improvement. NIG, Niguarda Hospital; BCH, Birmingham Women’s and Children’s Hospital; SEEG, stereo-EEG.*

We chose to limit the assessment of the tool to its performance in the analysis on a set of real data acquired in the context of the presurgical evaluation of patients with drug-resistant epilepsy. We therefore measured the spatial concordance between the iEEG electrodes with the highest HFO presence and the SOZ determined using the standard clinical evaluation including ictal and interictal iEEG, functional imaging when appropriate. The goodness of the latter was supported by the post-surgical outcome at 1 year. Other equally important aspects of the assessment of software such as code quality, usability, and sustainability, and of the individual but invaluable user’s experience were outside of the scope of the present manuscript, as was measuring the performance of the automated method against human visual assessment.

#### Data Recording

For NIG patients, intracerebral stereo-EEG (SEEG) was recorded from intracranial multichannel/multi-contact electrodes (DIXI Medical, 5–18 contacts; 2 mm length, 0.8 mm diameter; leads 1.5 mm apart). The number of electrodes and the sites for implantation were decided according to anatomical and clinical data collected during the non-invasive phase of the evaluation and varied between 5 and 18 contacts per intracranial electrode (maximum number of 192 recording channels). Band-pass filter of 0.016–500 Hz was used. EEG signal was acquired continuously with a Neurofax EEG-1100 system (Nihon Koden, Tokyo, Japan) and sampled at 1 kHz with 16-bit resolution. Each channel was off-line re-referenced with respect to its direct neighbor (bipolar derivations with a spatial resolution of 3.5 mm) to cancel-out effects of distant sources that spread equally to both adjacent sites through volume conduction. When appropriate for diagnostic purpose, the iEEG signal was referenced to the average signal from two electrodes identified from anatomical and neurophysiological data to be located in the white matter. Two scalp EEG channels (Fz and Cz referenced to a mastoid electrode) and chin electromyogram were recorded in addition to SEEG for sleep staging. The simultaneous video-iEEG recordings lasted between 5 and 10 days.

For BCH patients, SEEG recordings from 128 contacts were obtained using a commercial video-EEG monitoring system (System Plus, Micromed, Italy). Data were acquired with band-pass filter of 0.016–1 KHz and sampled at 2 KHz. The remaining recording parameters were the same as those used in the NIG patients.

Five patients had intracranial strips or grids implanted. In these patients, platinum-iridium alloy electrode disks (Ad-Tech Medical Racine, WI, United States), 4 mm diameter arranged in a grid (max 8 × 8 array), strip (4 × 1 or 6 × 1), and/or a combination of these were used. Electrodes were placed in the subdural space via craniotomies and/or burr hole craniotomies as appropriate.

#### Data Pre-processing

An expert neurophysiologist reviewed iEEG recordings and annotated the beginning of the seizures, together with the SOZ, anatomically defined by all the contacts involved in the onset of each seizure.

For each patient, peri-ictal and interictal data were analyzed by one of the authors (Lucia Rita Quitadamo) blinded to the clinical information and to the results of the diagnostic work-up. Peri-ictal data consisted of two seizure episodes. An EEG segment containing 10 min before and 2 min after the electrographic onset of each seizure was extracted and was considered as the period of interest for HFOs identification. Interictal data were extracted from 12 min of iEEG signal collected during stage III NREM sleep of the second night of the monitoring period. This choice was driven by evidence suggesting that the maximum likelihood of capturing HFOs is during NREM sleep (Bagshaw et al., 2009). Data were resampled to 1024 Hz (linear interpolation) and then visually inspected to identify artifactual channels, which were disregarded in subsequent analyses. A bipolar montage between adjacent contacts located in the gray matter was built in case of SEEG electrodes, while contacts were referenced to average reference in case of grid data; signal was then filtered in the 80–250 Hz frequency band. The original epoch was finally segmented in 2-min long sub-epochs, which were used for further analyses. Resampling and FIR filtering were performed using the “pop_resample” and “pop_eegfiltnew” functions from the EEGLAB suite.

### HFOs Detection Algorithm

The theoretical approach behind the algorithm was described in a recent paper by our group (Quitadamo et al., 2018). Nonetheless, in this section we briefly summarize the innovative nature of the algorithm used for the detection of HFOs that constitutes the backbone of the whole EPINETLAB software.

After signal preprocessing and segmentation, discrete wavelet transform is computed on 1-s signal windows. The preliminary data showed high-specificity and sensitivity (96.03 and 81.94%, respectively) using complex Morlet transform (Quitadamo et al., 2018). For each channel and each window, the scalogram is first computed, representing the percentage energy of each wavelet coefficient and then, for each frequency bin, the algorithm computes the spectral kurtosis, which reflects the presence of transient activities in a signal and which can be used to identify signal properties in the frequency domain (Antoni, 2006). The distribution of spectral kurtosis over all frequencies and channels is then fitted against a set of known distributions available in the Matlab “Statistics and Machine Learning Toolbox” (e.g., normal, exponential, gamma, generalized extreme value, etc.). The distribution ranking first in terms of logarithmic likelihood is selected as representative of the kurtosis in that specific EEG segment and its mean and variance values are determined. A threshold on the kurtosis distribution is set as: thresh=mean + 3SD; channels are then ranked according to the total number of windows with kurtosis peaks over *thresh* value in a restricted number of frequencies. To reduce the number of channels to be submitted to further analysis, the median (Q2), and the 75th percentile (Q3) are computed from the distribution of the number of windows. The list of the channels with a number of windows >Q2 + (Q3 - Q2)/2 can be retained for further analysis [the term (Q3 - Q2)/2 is added to take into account the positive skewness of the distribution]. As a result of this process, only highly kurtotic channels are retained for HFOs detection process, reflecting high prevalence of transient activities and therefore candidate to represent the SOZ. This process allowed significant reduction of computational load as HFOs are detected on a subset of relevant channels instead that on the whole initial dataset. Finally, candidate HFOs are identified as events in which the power of the wavelet coefficients calculated over 3ms-long consecutive windows, exceeds the mean power in the whole 1 s window by 5 SD and for more than 20 ms. Events with power spreading over all the frequency bands (e.g., spikes) or over many channels (e.g., muscular activities) are discarded as potential artifacts.

### EPINETLAB Implementation

#### Functional Blocks

**FIGURE 1:**
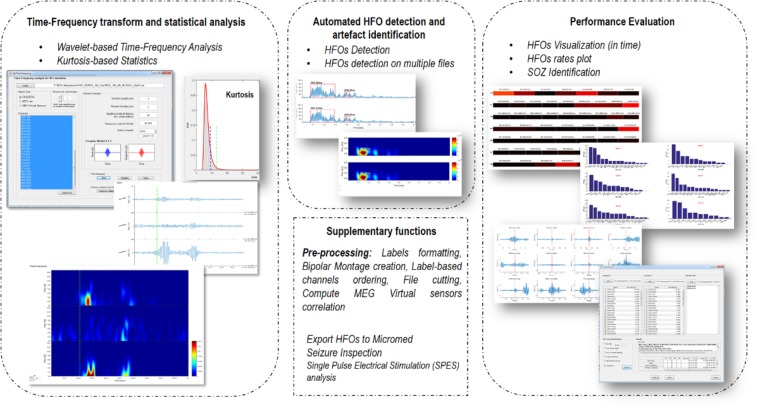
Functional blocks in EPINETLAB. Four different blocks of functions are defined: (1) Time-frequency transform and statistical analysis. (2) Automated HFO detection and artifact identification. (3) Performance evaluation. (4) Supplementary functions.

EPINETLAB is implemented as a collection of routines easily accessible from the EEGLAB main bar. Four different modules of functions can be recognized, as reported in **Figure 1**:

(1) *Time-frequency transform and statistical analysis*: these functions allow setting the parameters for the time-frequency analysis and to compute kurtosis-based statistical thresholding to identify the subset of channels with the highest occurrence of deviant activity, most probably associated with the presence of HFOs.
(2) *Automated HFO detection and artifact identification*: the detection of HFOs is performed on the power distribution of wavelet coefficients. The search can be done either on the whole channel pool or on a subset of these identified by means of kurtosis thresholding. Potentially artifactual events can be discarded based on quantitative criteria.
(3) *Performance evaluation*: detected HFOs can be graphed in both temporal and spatial domains. Moreover, the area with the highest HFO rate, which we hypothesize is related to the brain epileptogenic tissue, can be statistically determined according to different methodologies and compared to the clinically defined SOZ. This process is evaluated in terms of sensitivity and specificity of the detection.
(4) *Supplementary functions*: these functions allow to import MEG file, to compute correlation between MEG sources, to inspect seizure frequency content, to manipulate channel labels, montages, and file duration.

**FIGURE 2:**
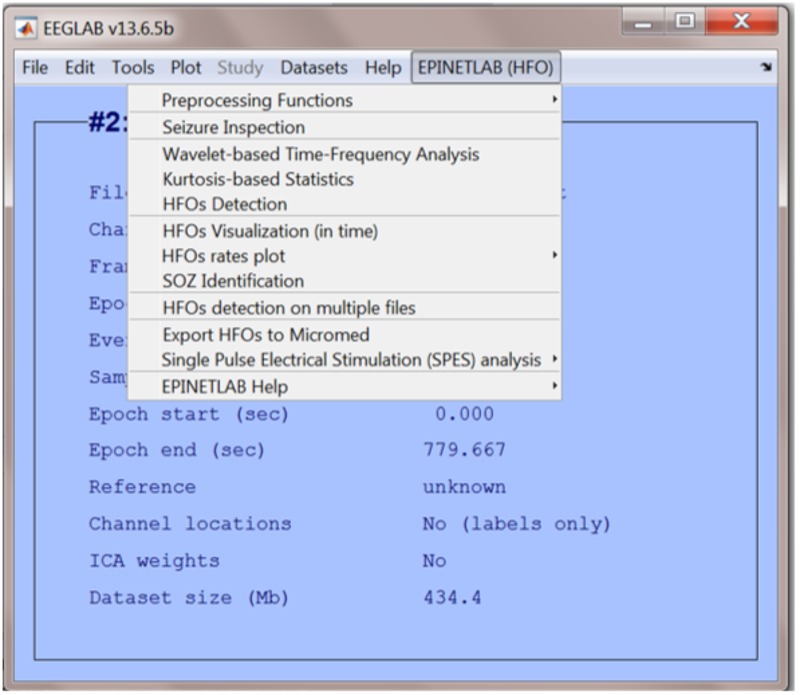
EEGLAB main bar with the EPINETLAB plugin installed.

When the EPINETLAB plugin is installed into the EEGLAB suite, a new tab (EPINETLAB (HFOs)) is created in the main EEGLAB bar menu, see **Figure 2**. As the tool makes use of the native EEGLAB file format (.set), naïve Matlab users can easily interact with the functionalities provided by both EEGLAB itself and EPINETLAB, so to exploit the generic signal pre-processing functions and the advanced HFO-recognition ones in a complementary modality.

#### Time-Frequency Analysis and Statistics

**FIGURE 3:**
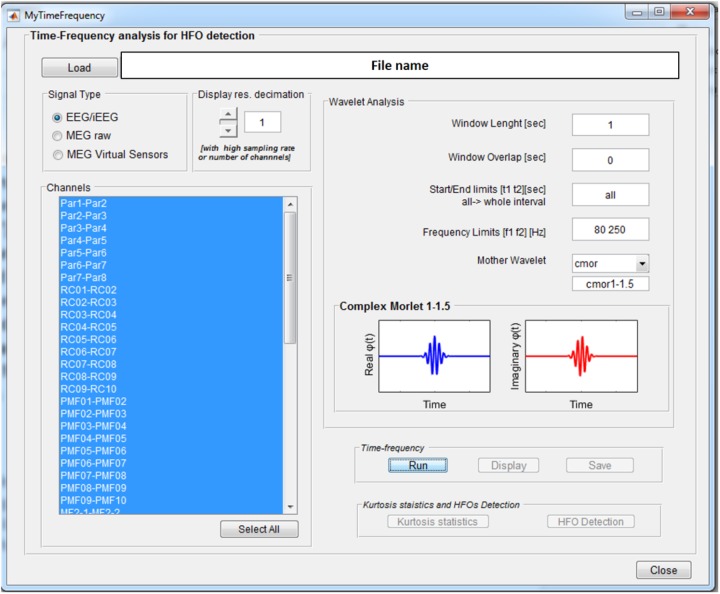
Time-frequency analysis GUI. Once an EEGLAB “.set” file is loaded, different parameters can be set up: channels on which to perform the analysis, length of the segmentation window (1 s), overlap between consecutive windows (0 s), signal time epoch to analyze (all), frequency band limits (80–250 Hz), and mother wavelet (Complex Morlet). Display resolution can be reduced in case of really high number of channels and/or sampling rate.

The *Wavelet-based Time-Frequency Analysis* functionality opens a GUI, see **Figure 3**, where the parameters needed to perform the wavelet-based time-frequency decomposition can be set.

These consist of the length of the window for signal segmentation, the overlap between consecutive windows, the signal time interval to be analyzed, the frequency band of interest, and the continuous mother wavelet used for the transform. All the wavelets available in the *Matlab Wavelet Toolbox* can be selected and are listed in the *wavelet display* panel, divided in real and imaginary parts in case of complex wavelets, as reported in **Figure 3**. A functionality is provided to reduce by a predefined factor the resolution of the time-frequency transforms to be displayed (see GUI below) in case of a high number of channels-high sampling rate combination. This does not affect signal properties but only the way data are displayed.

**FIGURE 4:**
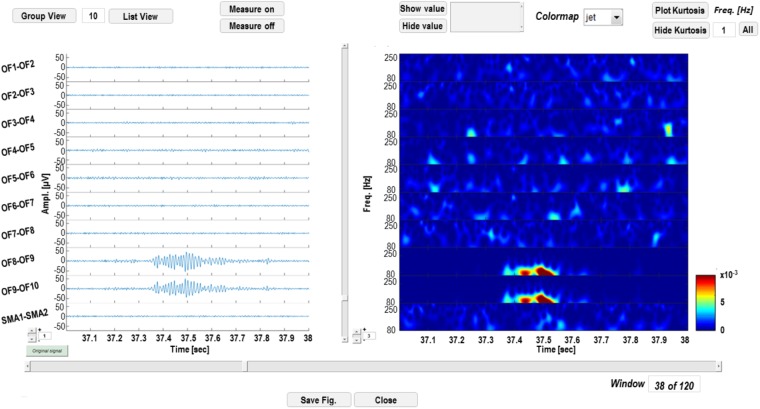
Time-frequency display GUI. In the left panel, signal in the time domain is displayed. Channels can be visualized in groups smaller than the original sample (“Group View” or “List View”) and amplitudes at different time instants can be measured, by enabling a cursor with the “Measure on” function. In the right panel, time-frequency transforms corresponding to the channels on the left are displayed and power of wavelet coefficients at different time instants or frequencies can be measured.

Wavelet-transformed signal can be saved into a Matlab (.mat) file and displayed as shown in **Figure 4**. The left panel is dedicated to time-domain signal visualization and measurement, whereas the right panel displays a time-frequency plot of the wavelet transforms. In the left panel, the user can scroll the signal within the windows defined by the segmentation; iEEG channels (and relative transforms) can also be displayed in smaller groups to improve the visualization of multichannel data and gain can also be adjusted to increase/reduce channels amplitude on screen. Signal voltage can be measured at user selected time instants by activating a cursor on the active screen (“Measure on” “Measure off”).

**FIGURE 5:**
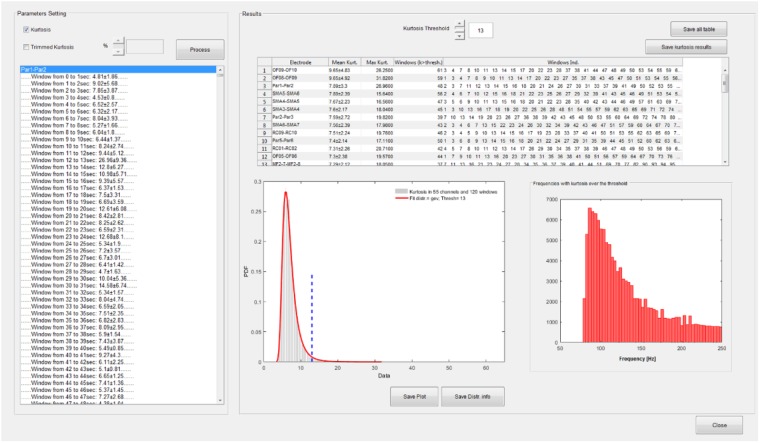
Statistical analysis GUI. Spectral kurtosis is computed on wavelet transforms in each segmentation window and averaged over frequencies. Kurtosis distribution is fitted with a set of known distributions available in the Matlab statistical toolbox. A kurtosis threshold is set on the distribution which equals the mean of the distribution plus 3 SD. Channels are ranked according to the number of windows with kurtosis over the threshold. Windows with kurtosis distributed uniformly over all the frequencies do not contribute to the ranking. In this case, the fitted distribution is a generalized extreme value (GEV) distribution and the threshold is equal to 13. In the upper right panel of the statistic GUI, the channels are sorted according to their mean kurtosis; in this case OF8–OF9 and OF9–OF10 are the channels with the highest number of windows with kurtosis exceeding 13. The distribution of frequencies with kurtosis over threshold is also depicted.

A functionality that performs the kurtosis-based statistical analysis on wavelet coefficients described in section HFOs Detection Algorithm is available from the time-frequency GUI (see **Figure 5**).

The distribution of spectral kurtosis of wavelet coefficients for all the segmentation windows is fitted and the kurtosis threshold (*thresh* value in section HFOs Detection Algorithm) is computed, see lower left panel in the right part of **Figure 5**. Channels are then ranked according to the largest number of windows with above-threshold kurtosis measure, see upper panel in right side of **Figure 5** and then the subset of significant channels can be selected as reported in section HFOs Detection Algorithm and saved in a text file for further analysis.

#### HFO Detection

**FIGURE 6:**
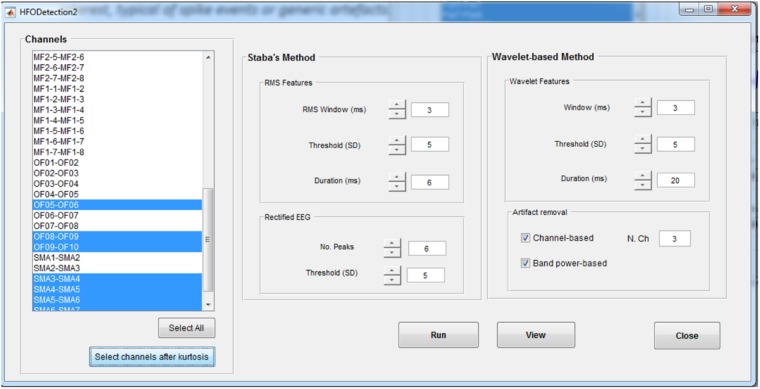
High-frequency oscillation detection GUI. This GUI allows to set up the parameters for the detection of HFOs with two different methods, the Staba and wavelet-based methods. Detection can be run on all the channels or on a subset of channels, determined, for example, from the kurtosis thresholding. For the wavelet-based analysis, a twofold artifact removal option is available: the channel-based one allows to reject candidate HFOs which synchronously spread on more than *N* channels (default value: 3); the band-power based one allows to reject candidate HFOs with power spreading uniformly over all the frequencies in the band of interest, typical of spikes.

In the *HFO Detection* GUI (**Figure 6**), the user can choose between the kurtosis-based method developed by our group (Quitadamo et al., 2018) and presented here and classical Staba detector (Staba et al., 2002). This has been used in the past as benchmark since it was the first algorithm to provide a semi-automatic detection of HFOs. The Staba detector computes the root mean square (RMS) of the signal in 3 ms windows and defines, as candidate HFOs, segments with RMS values at least five SD above the mean amplitude of the RMS signal and lasting more than 6 ms. The final condition to be met is the presence in the candidate HFO of at least six peaks greater than three SD from the mean value of the rectified band-pass signal.

The two implemented methods can be applied on all the channels or on a subset of them selected either manually or by the kurtosis-based thresholding as described in section HFOs Detection Algorithm. If using the kurtosis-based method, the detection of HFOs can be further refined by discarding the events that are occurring synchronously on multiple channels (potentially associated to the spreading of artifactual events) or with a power uniformly distributed over all the frequencies in the band of interest (potentially associated to HFO superimposed to spike events).

**FIGURE 7:**
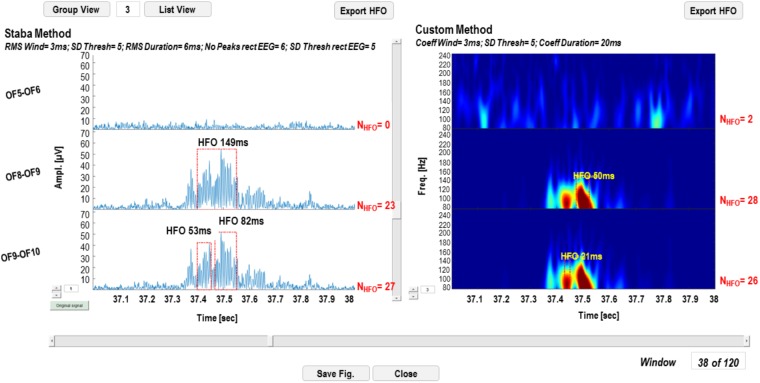
High-frequency oscillation detection display GUI. In the left panel, the results of the detection with the Staba detector are reported, while on the right panel results of the wavelet-based detection are reported. The total number of detected HFOs is reported next to each channel. The detected HFOs are indicated in the two panels with red dotted rectangles, while the duration of the event with a text string.

Detection results can be visualized on the original signal for the Staba detector and on the wavelet coefficients for the wavelet-based detector. The GUI in **Figure 7** is a simplified version of the one reported in **Figure 3**, except for the fact that, in the left panel, the rectified iEEG/MEG signal is reported, as required when using the Staba detector. To the right of each time series and each wavelet transform, the total number of detected HFOs is reported; the HFOs and their durations are indicated with red dotted rectangles and text strings in proximity. The list of all the detected events can be saved in two different text files, one for each detection modality. These text files are used for the evaluation and validation of the detection process.

**FIGURE 8:**
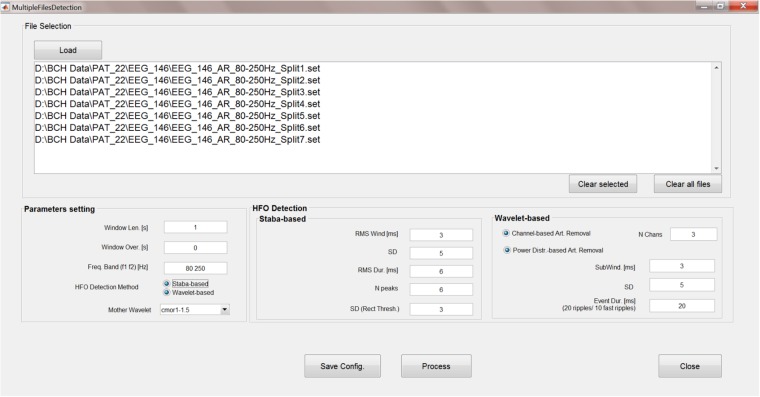
Multiple-file automatic HFO detection GUI. This GUI allows to run the time-frequency analysis, kurtosis thresholding, HFO detection, and artifact rejection in an automatic way and on multiple files. Results are saved in text files which can be used for further analyses.

The analyses steps described so far can be run sequentially, unsupervised and, more importantly, concurrently on multiple data files. A GUI has been created (**Figure 8**) to allow HFO detection by batch-processing all the chosen EEG files and to set the parameters for Staba and/or wavelet-based detection.

#### HFOs Rate Display

**FIGURE 9:**
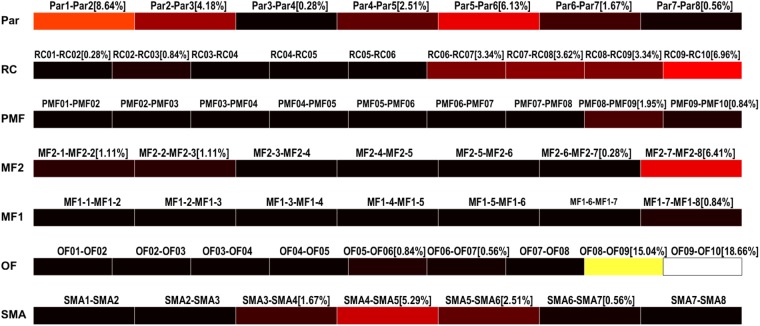
High-frequency oscillation rates display. HFO rates relative to each channel (bipolar, strips, or grid contacts) are visualized as colored bars. Percentages represent the number of HFO detected on the specific channel over the total number of detected HFOs. Color ranges from black, meaning HFO rate of 0%, to white, representing the maximum HFO rate. In this case, OF09–OF10 and OF08–OF09 are the channels with the highest HFO rates, 18.66 and 15.04%, respectively.

The results of the detection can be saved as multiple text files and used to display the HFO rates in each contact of each electrode as color-coded bars (**Figure 9**). Conventionally, the black color is associated to channels with no HFOs detected and the white represents the channel with the highest number of detected HFOs, normalized by the total amount of detected HFOs.

**FIGURE 10:**
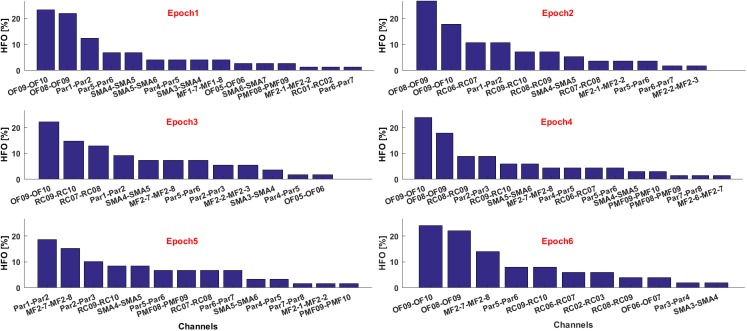
Contribute of the most informative channels to the overall HFO rate in six consecutive epochs.

If multiple text files are associated to consecutive time epochs on which the HFOs detection was performed, the contribution of each electrode in terms of HFO rate and relative to each epoch can be visualized as histograms, see **Figure 10**. This is useful, for example, to estimate the temporal evolution of the contribution of each channel to the generation of a seizure in terms of HFO rate.

The HFO rates of all contacts are automatically saved in a text file when the figures are created. Such files are then used in the subsequent steps leading to a probabilistic identification of the HFO area.

#### HFO Area Identification and Comparison With the SOZ

**FIGURE 11:**
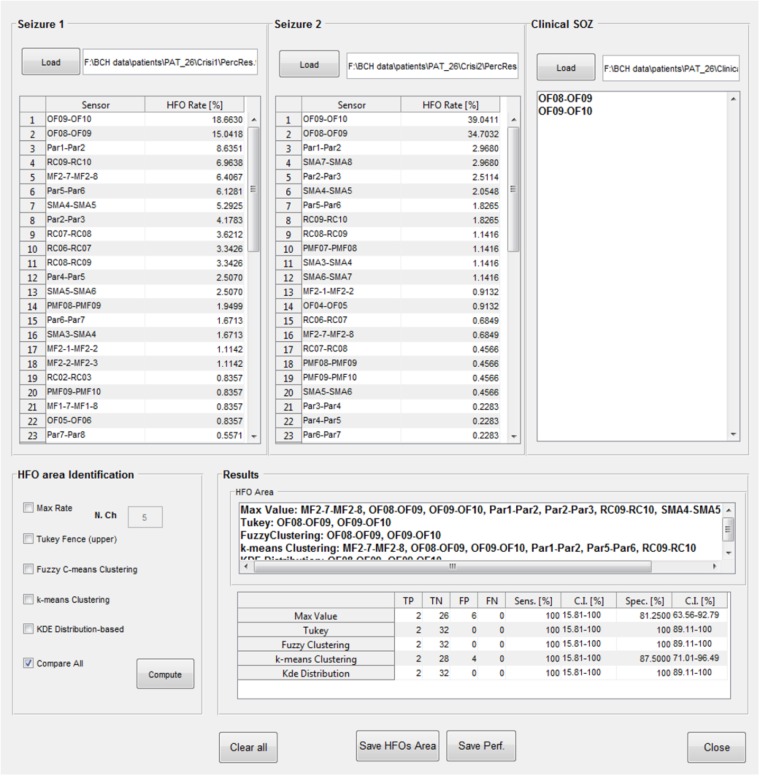
High-frequency oscillation area identification and comparison with the SOZ. Results of the detection of HFOs on all the analyzed channels and on two selected seizure episodes can be loaded, together with the SOZ identified by clinicians. Then five different algorithms (Max. Value, Tukey’s fence, fuzzy clustering, *k*-means clustering, and KDE distribution) select the contacts in the HFO area which can be then compared with the SOZ. Each method follows its internal statistical rule and can give different results. Results of the comparison between the HFO area and the SOZ are evaluated in terms of TP, TN, FP, FN, sensitivity, and specificity (with relative confidence intervals). In the case reported in figure, Tukey’s fence, Fuzzy clustering, and KDE-distribution methods achieve 100% specificity and sensitivity.

The identification of which channels belong to the “HFO area” is available in a further GUI (**Figure 11**); the results of the HFO detection process performed on up to two seizure episodes are loaded from text files produced at the end of the previous analysis step. A text file containing a list of contacts identified by the clinical team as belonging to the SOZ is then loaded. The channels attributed to the HFO area by EPINETLAB are investigated using five different methods:

(a) The “Max. Value” method: *N* electrodes with highest ranking in terms of HFO rate are selected as HFO area. In Quitadamo et al. (2018), we pragmatically chose *N* = 5 considering the typical spatial extension of the epileptic network in a focal epilepsy.
(b) The “Tukey’s fence” method: this method is typically used when outliers need to be identified in a dataset. Electrodes with an HFO rate higher than the upper Tukey’s fence (3rd quartile + 1.5 times the inter-quartile range) of all HFO rates, are selected. Channels within the HFO area can be considered data with anomalously high-HFO rates.
(c) The “Fuzzy c-means clustering” method (Jain et al., 1999): the method aims at identifying two natural clusters in the HFO rate dataset which the individual HFO rate belongs to with a defined degree of probability. Channels belonging to the cluster with the highest HFO rates and with a probability of belonging to that cluster higher than 0.7 are selected.
(d) The “*k*-means clustering” method (Jain et al., 1999): the selection procedure is similar to the one described above as point (c), but in which the cluster is identified using a *k*-means approach.
(e) The “Kernel Density Estimation (KDE)” method: a KDE (with Gaussian kernel) (Gliske et al., 2016) of the distribution of HFO rates is performed first. Then, after a smoothing procedure to reduce oscillations in the distribution, peaks, and troughs of the distribution are identified. If the value of the maximum peak exceeds by at least 1.8 times that of the closest trough, the value of HFO rate corresponding to the occurrence of the minimum trough of the HFO rate distribution is accepted as the threshold. The HFO rates exceeding that threshold are selected as HFO area.

Once the “HFO area” is identified, the software compares its spatial distribution with that of the electro-clinically defined SOZ. The performance of this comparison is listed in a table for each method in terms of true positives (TP, channels in the HFO area and in the SOZ), true negatives (TN, channels outside the HFO area and the SOZ), false positives (FP, channels in the HFO area but outside the SOZ), false negatives (FN, channels outside the HFO area but in the SOZ), sensitivity (TP/(TP + FN)), and specificity (TN/(TN + FP)), with relative confidence intervals. Results of the HFO area identification and comparison with the SOZ can be saved in text files.

#### Supplementary Functionalities

Additional functionalities, not strictly related to the detection of HFOs and the identification of the SOZ, are included in the present tool:

(1) Functions to import/export into/from EEGLAB an “.npx/.set” file into a “.set/.npx” (Bianchi et al., 2007). “Npx” is the native file format of the NPXLab suite (Bianchi et al., 2009), a framework for the analysis of EEG and brain-computer interface data.
(2) Functions to import into EEGLAB a MEG “.fif” file, native file format of the Elekta^®^ Neuromag TRIUX^TM^ system. Such functions are based on those found in the MNE toolbox (Gramfort et al., 2014). These functions were included to allow the analysis of electromagnetic recording within EPINETLAB.
(3) Functions to alphabetically order channel labels and to manipulate their label strings.
(4) Functions to reformat original data: users can create a bipolar montage from monopolar contacts calculating the potential difference between consecutive contacts of each SEEG electrode. This addition was deemed useful by clinicians as bipolar montages are generally preferred to unipolar in clinical SEEG interpretation to avoid the potential distortion of the intracranial signal from artefactual scalp EEG data or from positioning of the reference electrode in an active location. The average reference can also be computed and added as a “virtual” channel to the original dataset to allow reformatting the data against the average reference, a solution often used in the interpretation of intracranial subdural grids data.
(5) Functions to segment a file into *n* consecutive fixed-length epochs. This functionality is useful in the presence of large datasets which could cause significant computational burden in calculating wavelet transforms and spectral kurtosis on lower-spec PCs.
(6) Functions to inspect the time-frequency content of an iEEG epoch. This function was inspired by an extremely useful feature of Elpho-SEEG, a Labview software which allows to evaluate frequency distribution of EEG signal over time (Gnatkovsky et al., 2011).
(7) Functions for the single pulse electrical stimulation analysis (Klooster et al., 2011).
(8) Functions to export detected HFOs in Micromed-compatible format [Micromed s.p.a, Mogliano Veneto (TV), Italy]. This utility allows users of Micromed EEG systems to superimpose detected HFOs on a Micromed EEG file. Clinicians can integrate the results of the released automated algorithm with a more familiar clinical environment.

## Results

EPINETLAB underwent a 6-month extensive beta-testing program by experts from a wide range of backgrounds (engineers, medical doctors, clinical physiologists, and EEG technicians), in order to refine the user interface and minimize program crashes.

In the 12 patients of the validation set, the detection of the HFO area with the Tukey’s fence method and the *k*-means clustering, which has provided the best performance in a recent study by our group (Quitadamo et al., 2018), was evaluated in comparison with the clinically identified SOZ; results are reported for each patient in **Table 2** for peri-ictal data and in **Table 3** for interictal data.

**Table 2:** Results of the validation of the HFO detection and HFO area identification algorithm in peri-ictal epochs, by comparison with the clinically defined SOZ, for 12 representative subjects.

Patient	Method	TP	TN	FP	FN	Sensitivity (%)	CI (%)	Specificity (%)	CI (%)
1	Tukey	3	86	3	3	50	11.81–88.19	96.63	90.46–99.3
	*k*-means	4	71	17	2	66.67	22.28–95.67	80.68	70.88–88.32
2	Tukey	3	101	4	0	100	29.24–100	96.19	90.53–98.95
	*k*-means	3	92	13	0	100	29.24–100	87.62	79.76–93.24
3	Tukey	5	75	4	0	100	47.82–100	94.94	87.54–98.6
	*k*-means	5	78	1	0	100	47.82–100	98.73	93.15–99.97
4	Tukey	4	76	3	2	66.67	22.28–95.67	96.20	89.3–99.21
	*k*-means	4	77	2	2	66.67	22.28–95.67	97.47	91.15–99.69
5	Tukey	3	83	7	1	75	19.41–99.37	92.22	84.63–96.82
	*k*-means	3	89	1	1	75	19.41–99.37	98.89	93.96–99.97
6	Tukey	2	32	0	0	100	15.81–100	100	89.11–100
	*k*-means	2	28	4	0	100	15.81–100	87.50	71.01–96.49
7	Tukey	2	17	1	1	66.67	9.43–99.16	94.44	72.71–99.86
	*k*-means	3	15	2	0	100	29.24–100	88.24	63.56–98.54
8	Tukey	1	20	0	1	50	15.81–100	84.21	60.42–96.62
	*k*-means	1	17	3	1	50	1.26–98.74	85	62.11–96.79
9	Tukey	5	23	1	1	83.33	35.88–99.58	95.83	78.88–99.89
	*k*-means	6	20	3	0	100	54.07–100	86.96	66.41–97.22
10	Tukey	2	23	1	2	50	6.76–93.24	95.83	78.88–99.89
	*k*-means	2	18	6	2	50	6.76–93.24	75	53.29–90.23
11	Tukey	3	16	2	4	42.86	9.9–81.59	88.89	65.29–98.62
	*k*-means	3	15	3	4	42.86	9.9–81.59	83.33	58.58–96.42
12	Tukey	2	22	6	1	66.67	9.43–99.16	82.14	63.11–93.94
	*k*-means	3	17	10	0	100	29.24–100	62.96	42.37–80.6

**Table 3:** Results of the validation of the HFO detection and HFO area identification algorithm in interictal epochs, by comparison with the clinically defined SOZ, for 12 representative subjects.

Patient	Method	TP	TN	FP	FN	Sensitivity (%)	CI (%)	Specificity (%)	CI (%)
1	Tukey	3	57	5	3	50	11.81–88.19	91.94	82.17–97.33
	*k*-means	4	36	25	2	66.67	22.28–95.67	59.02	45.68–71.45
2	Tukey	3	59	1	0	100	29.24–100	98.33	91.06–99.96
	*k*-means	3	46	14	0	100	29.24–100	76.67	63.96–86.62
3	Tukey	2	54	0	3	40	5.27–85.34	100	93.4–100
	*k*-means	5	40	11	0	100	47.82–100	78.43	64.68–88.71
4	Tukey	1	26	0	5	16.67	0.42–64.12	100	86.77–100
	*k*-means	4	17	6	2	66.67	22.28–95.67	73.91	51.59–89.77
5	Tukey	3	44	2	1	75	19.41–99.37	95.65	85.16–99.47
	*k*-means	4	34	11	0	100	39.76–100	75.56	60.46–87.12
6	Tukey	2	20	0	0	100	15.81–100	100	83.16–100
	*k*-means	2	15	5	0	100	15.81–100	75	50.9–91.34
7	Tukey	1	25	3	2	33.33	0.84–90.57	89.29	71.77–97.73
	*k*-means	1	25	3	2	33.33	0.84–90.57	89.29	71.77–97.73
8	Tukey	1	24	1	1	50	1.26–98.74	96	79.65–99.9
	*k*-means	2	18	6	0	100	15.81–100	75	53.29–90.23
9	Tukey	1	22	2	5	16.67	0.42–64.12	91.67	73–98.97
	*k*-means	2	14	9	4	33.33	4.33–77.72	60.87	38.54–80.29
10	Tukey	2	15	2	2	50	6.76–93.24	88.24	63.56–98.54
	*k*-means	2	14	4	2	50	6.76–93.24	76.47	50.1–93.19
11	Tukey	2	9	1	5	28.57	3.67–70.96	90	55.5–99.75
	*k*-means	6	4	2	1	85.71	42.13–99.64	66.67	22.28–95.67
12	Tukey	1	11	3	2	33.33	0.84–90.57	78.57	49.2–95.34
	*k*-means	3	6	6	0	100	29.24–100	50	21.09–78.91

In peri-ictal epochs, Tukey’s method resulted in average sensitivity of 70.93% and average specificity of 93.12%, while *k*-means clustering with average sensitivity of 79.27% and average specificity of 80.03%. Using the Youden’s metric of overall system performance (Youden, 1950) (J = sensitivity + specificity - 1, with sensitivity and specificity expressed as unit fraction), an index value of 0.64 is obtained for Tukey method and of 0.59 for *k*-means method.

In interictal epochs, Tukey’s method resulted in average sensitivity of 49.46% and average specificity of 93.30%, while *k*-means clustering had an average sensitivity of 77.97% and average specificity of 71.40%. A Youden’s index value of 0.43 is obtained for Tukey method and of 0.49 for *k*-means method.

## Discussion

This manuscript presents a Matlab toolbox developed with the aim of supporting researchers and clinicians in the detection of HFOs and the identification of the SOZ in iEEG/MEG data. The tool was designed as a plugin of the EEGLAB framework, a widely used, user-friendly software for the analysis of brain electromagnetic data. The plugin is released for free upon request under the limitations of the GNU GPL 3.0.

EPINETLAB was implemented as a multi-GUI set of functions to allow users not experienced in the Matlab environment to apply advanced signal processing techniques to datasets acquired during pre-surgical evaluation. The tool is based on a structured analysis pipeline and allows to pre-process data, compute time-frequency transformation of EEG signal, operate a kurtosis-based selection of the most informative channels, which was the most innovative aspect of the algorithm released by our group, detect HFO events, reject artifact events, and finally identify the “HFO area” using appropriate and robust statistical testing. Moreover, a functionality for the statistical comparison of the HFO area with the clinically defined SOZ is provided and the process is evaluated in terms of TP, FP, TN, FN, sensitivity, and specificity.

The preliminary validation of this tool on a small group of patients who were investigated with iEEG using a combination of grid/strips and SEEG and successfully operated showed good concordance between electrodes with the highest contribution of HFOs and the SOZ identified clinically. The analysis suggests that, at least in this subset of patients, peri-ictal segments of iEEG offer a better yield than those selected in the interictal state during sleep. This finding requires further validation on larger patient cohorts and this toolbox can facilitate large-scale data analysis, removing bias due to inherent subjectivity, and lack of quantitative measures associated with visual inspection of the iEEG trace.

In our opinion, the main strengths of the toolbox are:

• The compatibility with the most used file formats for brain data, which favors sharing of data and the dissemination of results.
• The possibility to import and analyze MEG data, which allows to compare results on the SOZ from complementary methodologies.
• The GUI-oriented approach used for the software implementation, which allows also non-specialist users to easily set parameters and independently run the analysis.
• The totally automated HFO detection process, which decreases unavoidable human bias in case of high-density, long term recordings.
• The possibility to modify the code and extend its functionalities, adding new wavelet transforms or new algorithm for the detection, for example, as source-code is released.

The final version of the tool ready for release incorporates improvements that resulted from the feedback received during extensive beta testing by different professional groups in three departments. With modest training the tool can be used by professionals who are conversant with properties of neurophysiological signals. HFOs detection and SOZ identification are a topic of great interest at this time in epilepsy practice and research and we developed this tool hoping that it will constitute a valid support for clinicians who are currently tasked with visual analysis of iEEG.

Finally, this software in its current implementation is intended to provide only a limited 2D graphic representation of the electrodes; the contribution of each contact to the total HFO content of the analyzed signal is color-coded as seen in **Figure 9**. However, quantitative data are exported in ASCII format and can be used to create objects in existing image-fusioning software such as 3D-Slicer (Fedorov et al., 2012).

## Conclusion

A novel user-friendly and multi-GUI EEGLAB plugin is implemented for the detection of HFOs and the identification of the SOZ, according to an innovative algorithm already clinically validated and released by or group within an EU-funded project. It provides clinicians with a set of GUI-based and user-friendly functionalities that can be available to research teams working on epilepsy presurgical workup data.

## Data Availability Statement

The datasets analyzed in this study will be available from the corresponding author upon reasonable request. EPINETLAB is released under the GPL version 3 License. Code is available at https://github.com/quitadal/EPINETLAB.

## Ethics Statement

This study was a retrospective/secondary analysis of anonymized data obtained in the context of standard clinical practice; as such it did not require retrospective consent and was authorized by the Comitato Etico Area C, Ospedale Niguarda Ca’ Granda (14.7.2014), and the R&D Department of the Birmingham Children’s Hospital NHS Foundation Trust (ref. APGE14 14.10.2014).

## Author Contributions

LQ designed, coded, and tested EPINETLAB. EF provided MEG data and performed the analysis. RM, LdP, and NS provided iEEG data and validated results as clinical experts. SS contributed to the conceptual stage of the software development, provided iEEG data, performed the analysis, and validated results as a clinical expert. All the authors read, reviewed, and approved the manuscript.

## Conflict of Interest Statement

The authors declare that the research was conducted in the absence of any commercial or financial relationships that could be construed as a potential conflict of interest.

## Footnotes

1 http://cordis.europa.eu/project/rcn/195032_en.html
